# Venular amyloid accumulation in transgenic Fischer 344 Alzheimer’s disease rats

**DOI:** 10.1038/s41598-022-19549-y

**Published:** 2022-09-10

**Authors:** J. Bishay, T. L. Beckett, A. Y. Lai, M. E. Hill, D. McMahon, J. McLaurin

**Affiliations:** 1 Biological Sciences, Sunnybrook Research Institute, Toronto, ON M4N 3M5 Canada; 2 Physical Sciences, Sunnybrook Research Institute, Toronto, ON M4N 3M5 Canada; 3 Laboratory Medicine and Pathobiology, Temerty Faculty of Medicine, University of Toronto, 2075 Bayview Avenue, Rm S111, Toronto, ON M5S 1A8 Canada

**Keywords:** Neuroscience, Alzheimer's disease, Mass spectrometry

## Abstract

Strong evidence demonstrates a significant association between cerebral amyloid angiopathy (CAA) and Alzheimer’s disease (AD). For this reason, interest in understanding the underlying vascular pathologies that contribute to AD remain. CAA research has primarily focused on arterioles and capillaries, overlooking the draining venules. Therefore, this study sought to examine venular amyloid pathology and its relationship to arteriolar amyloidosis throughout AD progression in the TgF344-AD rat model. Antibodies targeting the amyloid-beta peptide (Aβ) sequence suggest morphological differences between arteriolar and venular amyloid. Mass spectrometric analyses of isolated cortical parenchymal plaques, arteriolar and venular amyloid demonstrated presence of Aβ in all three samples, as well as proteins known to be associated with AD. Histopathological analysis indicates a significant age effect for both arteriolar and venular amyloid accumulation, with accumulation initiated in the somatosensory cortex followed by the motor and cingulate cortex. Lastly, significant arteriolar amyloid accumulates relative to venular amyloid deposition in AD progression. Overall, understanding venular and arteriolar amyloid pathology provides insight into the complex connection between CAA and AD.

## Introduction

With Alzheimer’s disease (AD) being the most common form of dementia in the elderly^1^, there has been an overwhelming drive in research to understand the intricacies of this complex neurodegenerative disease for the discovery of therapeutics and treatment. AD is characterized by several clinical symptoms including cognitive decline, memory impairment, and loss of activities of daily living^1–3^. The underlying mechanisms of this disease is distinctly marked by the accumulation of two proteins; extracellular amyloid-beta peptides (Aβ) and intracellular hyperphosphorylated tau^2,3^. The build-up of Aβ results in the formation of amyloid plaques spreading through the cortex then into the hippocampus^4,5^, leading to neuronal death and brain atrophy^6,7^. Aβ aggregates also build up in the walls of the vasculature, a pathology known as cerebral amyloid angiopathy (CAA)^8–10^. CAA is a small vessel disease defined as the accumulation of various amyloid proteins, leading to cerebrovascular dysfunction and severe clinical symptoms including stroke, intracerebral hemorrhages, and dementia^6,8,11^. Despite it being a distinct pathology^12^, CAA is highly comorbid with AD, with prevalence of 80–90% in AD patients^9,10^. Due to their highly integrative nature, there has been a need to investigate the role of the cerebrovasculature and of CAA to understand how the vasculature contributes to AD.

The venous system of the cerebrovasculature, until recently, has been under-studied in CAA and, in association, Alzheimer’s Disease (AD)^13^. There has been increased interest in studying the pathology of veins and venules, not only in the context of CAA, but also in dementia^3,4,14–16^. Venous pathology has been shown to contribute to vascular dysfunction in AD, resulting in white matter hyperintensities, microinfarcts, and potentially induction of ischemia^14,16,17^. Furthermore, pathological studies showed the presence of amyloid not only in arterioles, but also in venules of 21 patients with one case having severe amyloid deposition in venules^18^. Weller and colleagues demonstrated amyloid-beta peptide (Aβ) deposition in venules, although to a much lesser extent compared to arteriolar Aβ deposition^19,20^. These early studies were strengthened by pilot data showing that retinal venular tortuosity and amyloid burden correlate with verbal memory loss in patients^15^, and renewed interest in venular contributions to disease progression.

Current rodent disease models suggest that even when rare, venular Aβ impaired glymphatic clearance, contributed to Aβ deposition, and exacerbated CAA and AD progression^1,3,21,22^. In the APP + PS1 rat model, pathological examination demonstrated Aβ deposits in leptomeningeal and cortical arterioles, cortical capillaries, as well as the leptomeningeal and cortical venules^22^. In addition, there were significant venular alterations including enlarged cortical venules and perivascular spaces, with stenosis consisting of collagen, tau, and Aβ^22^. Further studies identified Aβ in venules of TgF344-AD rats, a model that overexpresses human Swedish amyloid precursor protein (APPswe) and presenilin 1 with exon 9 excised (PS1ΔE9)^1,23^. Joo et al., demonstrated that vascular Aβ deposition was not restricted to arterioles, being observed in venules at 9 months of age. This study also found a 51% decrease in vascular reactivity in venules in response to hypercapnic challenge^1^. These previous studies demonstrate the presence of venular Aβ, however, the temporal link between arteriolar and venular Aβ remains unclear. The present study investigated the pathological characteristics of venular amyloid and characterized the interplay between arteriolar and venular amyloid deposits as a function of aging and AD progression. The TgF344-AD rats were used in this study as they demonstrate early neurovascular dysfunction that contributes to CAA^1^ and recapitulates progressive vascular amyloidosis^23^. These rats accumulate venular amyloid, previously reported in AD patients^18,24,25^. Herein we show distinct morphological characteristics between venular and arteriolar amyloid and that venular amyloid accumulates significantly with disease progression, but to a lesser degree of deposition compared to arteriolar amyloid.

## Results

### Morphological differences between arteriolar and venular amyloid

**Figure 1:**
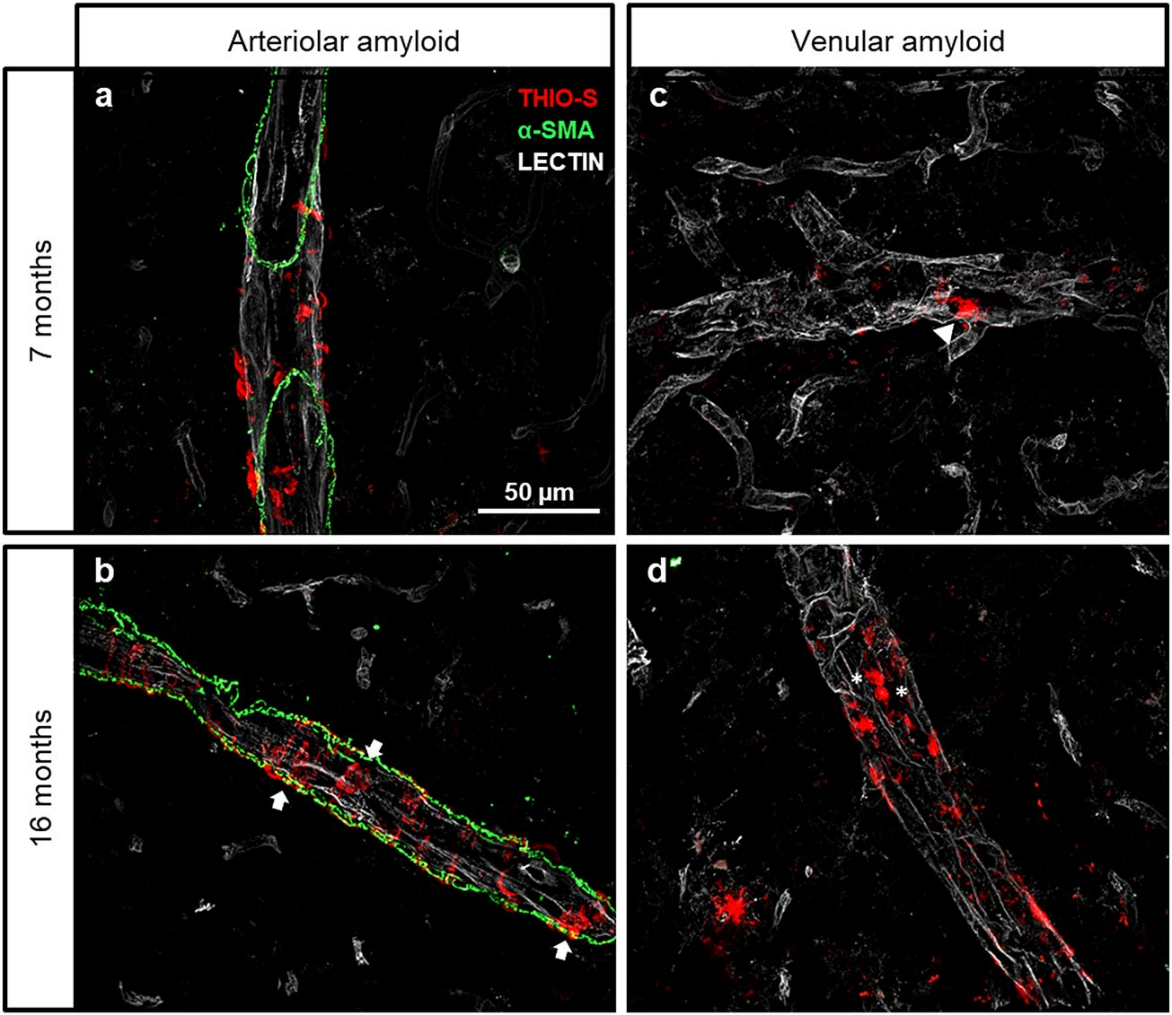
Morphology of ThioS-positive vascular amyloid in arterioles and venules with disease progression. Vascular amyloid was stained with ThioS (red), which targets proteins rich in β-sheet structures, at 7 months and 16 months of age. Arteriolar amyloid at 7 months (**a**) demonstrates small amyloid deposits that progressed to a cyclical ring-like structure (white arrows) surrounding the arterioles at 16 months (**b**). Arterioles (**a**, **b**) are distinguished from venules (**c**, **d**) by labelling with alpha smooth muscle actin (green). Venular amyloid deposits remained globular in shape (white triangle) from 7 months (**c**) to 16 months of age (**d**), with presence of deposits appearing cellular in shape (white asterisks). Images were acquired with a 40 × objective.

Previous literature suggests that vascular amyloid deposits are not exclusive to cerebral arterioles^1,22^. We sought to characterize the amyloid deposits in TgF344-AD rats using histopathology with amyloid dyes and Aβ-specific antibodies and to confirm Aβ within vascular amyloid using laser capture microscopy and mass spectrometry. Thus, we first set out to characterize the Aβ deposits in the TgF344-AD rat model of Alzheimer’s disease across disease progression (Fig. 1). We demonstrate both arteriolar and venular amyloid deposits associated with vessel walls that visually increase in volume with disease progression. The arteriolar amyloid deposits demonstrate globular deposits at 7-months of age that progressed to the classical ring-type structures surrounding arterioles by 16 months of age (Fig. 1a, b). In contrast, venular amyloid deposits resembled globular arteriolar amyloid at 7-months of age and remained globular even as disease progressed (Fig. 1c, d). An increase in the number of globular deposits and the size of these deposits was observed as disease progressed (Fig. 1c, d). Moreover, ~ 12% and ~ 20% of the Thioflavine S (ThioS)-positive amyloid deposits appeared cellular in shape and were localized within the lectin-stained venules at 7 months and 16 months of age, respectively. This is consistent with a previous report that demonstrated monocytes surrounding Aβ-laden venules^10^.

**Figure 2:**
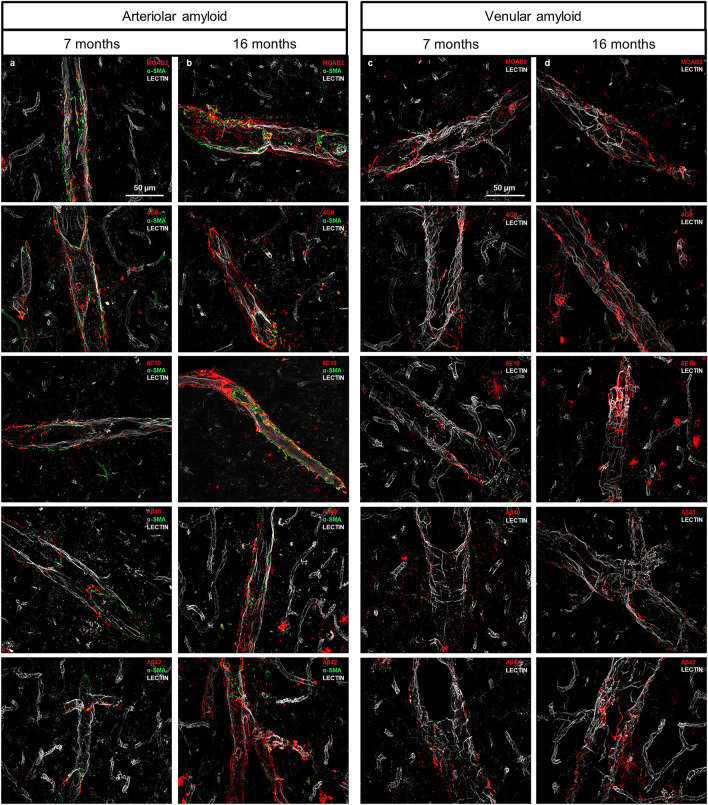
Arteriolar and venular amyloid stained with Aβ antibodies to visualize vascular Aβ. Aβ antibodies MOAB-2, 4G8, 6E10, Aβ_40_, and Aβ_42_ (red) target specific amino acid regions of the Aβ peptide sequence, allowing for a comprehensive visualization of vascular Aβ. Arteriolar Aβ initially deposits around alpha smooth muscle actin (green), labelling the arterioles, at 7 months of age (**a**), while amyloid circles the arterioles at 16 months of age (**b**). An increase was also shown in venular Aβ, with globular deposits increasing in size and number between 7 (**c**) and 16 months of age (**d**). Images were obtained at 40 × objective.

Amyloid has been characterized using structure-based dyes, including Congo red, ThioS and methoxy-X04, which label three-dimensional β-sheet structured proteins including but not restricted to Aβ^1–3,18,26^. To visually identify the presence of Aβ, arteriolar and venular amyloid throughout the cortex was immunolabeled with five anti-Aβ antibodies covering distinct epitopes across the entire Aβ sequence; MOAB-2 [epitope: amino acids 1-4], 6E10 [epitope:1-16], 4G8 [epitope:17-24], Aβ_40_ [epitope:x-40], and Aβ_42_ [epitope: x-42]. Cortical regions, including the cingulate, motor, somatosensory, secondary auditory, and insular cortices were investigated at 7 months (Fig. 2a, c), 10 months, 13 months (Supplementary Fig. S1), and 16 months of age (Fig. 2b, d). With anti-Aβ antibodies, positive immunolabeling is dependent on the availability of the primary sequence of Aβ, thus an antigen retrieval step was performed. For this reason, the morphology of the vascular deposits identified with anti-Aβ antibodies is distinct from amyloid stains, such as ThioS. In arterioles, ThioS identified thick sheets of amyloid circling the lectin-stained vessels, whereas, Aβ immunolabeled with the anti-Aβ antibodies were thinner and spread throughout the arterioles, particularly along and outside the edge of the vessel, where the perivascular spaces are located. There was a noticeable increase in arteriolar amyloid with disease progression after visualization with 4G8 and 6E10 (Fig. 2a, b). In addition, Aβ visualized using the N-terminus specific antibody, MOAB-2, were dotted around the arterioles, mostly compressed around alpha smooth muscle actin at 7 months of age (Fig. 2a), that progressively circled the arteriole at 10, 13 (Supplementary Fig. S1), and 16 months of age (Fig. 2b). As disease progresses, there was noticeably more Aβ_42_- than Aβ_40_- positive staining (Fig. 2a, b). In venules, all antibodies stained Aβ as globular shaped deposits, that were smaller than those detected with ThioS, and adhered to the edge of the vessel (Fig. 2c, d). Analyzing MOAB-2, 4G8, and 6E10 immunolabeling, there was an increase in fluorescence intensity from 7 to 16 months of age, where the globular deposits became larger in size (Fig. 2c, d). As was detected for arterioles, venular amyloid demonstrated noticeably more Aβ_42_ immunolabeling than Aβ_40_, suggesting that Aβ_42_ was preferentially deposited in venules over Aβ_40_. Alternatively, Aβ_40_ is known to bind preferentially to vascular amyloid^9,27,28^, however, TgF344-AD rats overexpress APP containing the Swedish mutation and PS1ΔE9, both of which increase the production of Aβ_42_ over Aβ_40_.

### The Aβ peptide and other AD-associated proteins were identified in venular amyloid

To confirm the presence of Aβ peptides within venular deposits, a combination of the laser capture microscopy and mass spectrometry was utilized. We collected enriched samples of parenchymal, arteriolar, and venular amyloid deposits, from 12- and 16-month-old TgF344-AD rats^23^. Cortical plaques were isolated from the motor, somatosensory, auditory, piriform, entorhinal, and ectorhinal cortices, while arteriolar and venular amyloid were obtained from the cingulate, motor, somatosensory, secondary auditory, and insular cortices. We pooled 1600 parenchymal, 1900 arteriolar and 7700 venular amyloid samples for the mass spectrometry experiments. The difference in the number elements isolated for each amyloid sample is a result of the difference in deposit size, with parenchymal plaques being more dense and much larger than vascular amyloid. Meanwhile, venular amyloid forms smaller, globular deposits compared to arteriolar amyloid (Fig. 1) and thus the necessity for increased elements to accommodate detection limits of the mass spectrometer. Proteomic analysis was conducted on the Thermo Scientific Orbitrap Fusion Lumos Tribrid Mass Spectrometer containing Quadrupole Technology with an ultrahigh resolving power for increased accuracy and specificity towards proteins/peptides of small molecular sizes. The total spectrum counts from each sample identified Aβ (Table 1). Three Aβ peptide fragments that were detected: human Aβ sequence (DAEFRHDSGYEVHHQK), rat Aβ sequence (DAEFGHDSGFEVR), and human and rat Aβ sequence (LVFFAEDVGSNK) (Table 1). Previous literature has demonstrated in vivo that rat Aβ co-aggregates with human Aβ, which is observed in these plaque samples^29^. Overall, the data demonstrates Aβ to be more abundant in the plaques and arteriolar amyloid compared to venular amyloid.

**Table 1:** Total spectrum counts of the Aβ peptide in parenchymal plaques, arteriolar and venular amyloid samples.

Peptide sequence	Cortical tissue CTRL	Parenchymal plaques	Arteriolar tissue CTRL	Arteriolar amyloid	Venular tissue CTRL	Venular amyloid
DAEFRHDSGYEVHHQK (HUMAN)	–	3	–	2	–	–
LVFFAEDVGSNK (RAT/HUMAN)	1	4	1	3	1	2
DAEFGHDSGFEVR (RAT)	–	1	–	–	–	1
Total	1	8	1	5	1	3

Several classes of proteins were identified in the amyloid samples (Supplementary Tables S1–S3), many previously reported within amyloid plaques^30,31^. Protein classes included cytoskeletal proteins, (tubulin, actin, and microtubule-associated proteins) and membrane trafficking-associated proteins (synaptosomal-associated proteins, vesicle-associated membrane proteins, dynein, and clathrin). Among this class of proteins, dynamin 1 was previously reported to be enriched in a mouse model of AD^30^ and AD patients^31^. Glial fibrillary acidic protein, a specific marker for astrocytes, was present in both parenchymal plaque and arteriolar amyloid samples (Supplementary Table S1 and S2). Since activated astrocytes are known to surround amyloid plaques and blood vessels^32^, the colocalization supports the integrity of the samples. Interestingly, ApoE was present in the parenchymal plaque, arteriolar and venular amyloid samples (Supplementary Tables S1–S3), which further supports the relationship between ApoE and AD^6,9,33–35^. Among the proteins identified exclusively in the venular amyloid sample, collagen was identified, specifically, collagen α-1(I) (Supplementary Table S3). In summary, tandem mass spectrometry analysis of the laser capture microdissection-derived amyloid samples identified a comprehensive list of proteins associated with parenchymal plaques, arteriolar and venular amyloid (Supplementary Tables S1–S3).

### Venular amyloid accumulation proceeds significant arteriolar amyloid deposition

**Figure 3:**
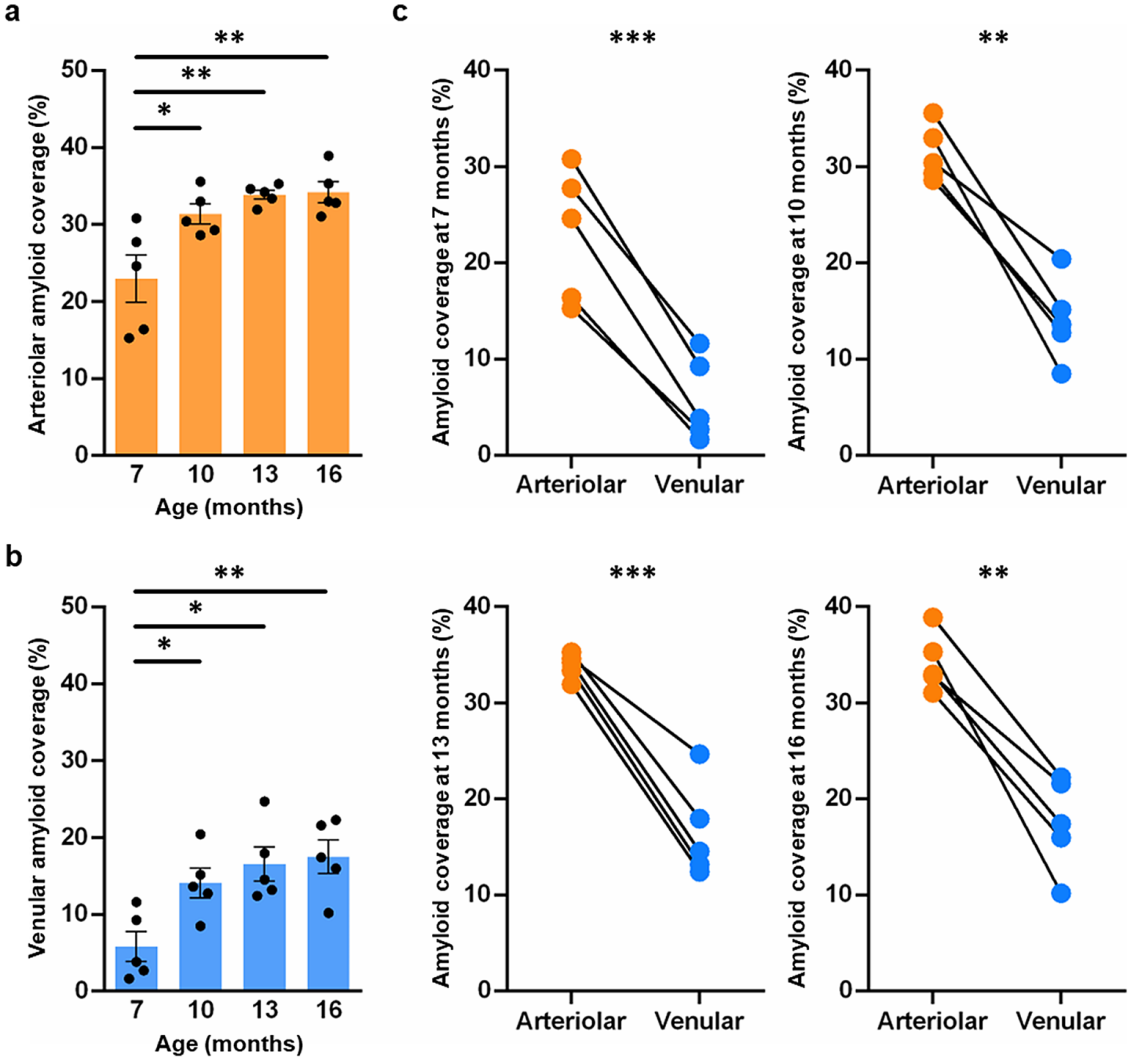
Percent arteriolar and venular amyloid load with disease progression. Percent vascular amyloid coverage was determined by quantifying area of ThioS over area of lectin-labelled vessels. Arteriolar (**a**; p = 0.0001**)** and venular amyloid (**b**; p = 0.004**)** increases significantly with disease progression. The majority of amyloid increased between 7 and 10 months of age for both arteriolar (p = 0.02) and venular amyloid (p = 0.05). Using two-tailed t-tests statistical approach, the scatter plot comparing percent amyloid load between arterioles and venules (**c**) demonstrates significant arteriolar amyloid relative to venular amyloid within each individual rat: 7 (p = 0.006), 10 (p = 0.021), 13 (p = 0.0008), and 16 months of age (p = 0.019). Alpha = 0.05. Graphs are labelled as the mean of five replicates ± sem. (*p ≤ 0.05, **p ≤ 0.01, ***p ≤ 0.001, ****p < 0.0001).

**Figure 4:**
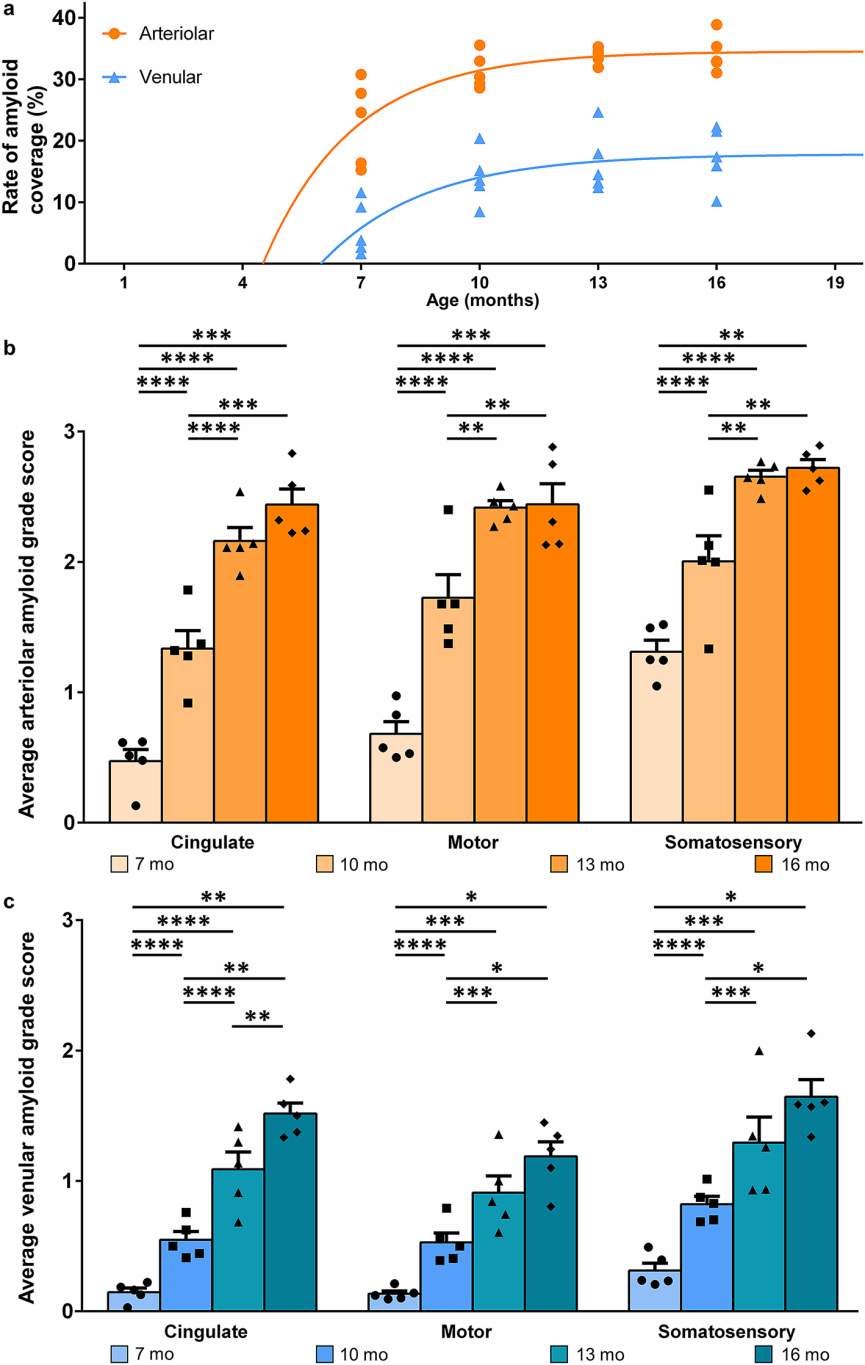
Rate of vascular amyloidosis and severity in AD. A one-phase association model was utilized to compare the accumulation rate of arteriolar and venular amyloid with AD progression (**a**). Arteriolar amyloid (orange, k = 0.4389) accumulated at a faster rate than venular amyloid (blue, k = 0.3860). Vascular amyloid severity was categorized based on an established grade scoring system (0–4)^36^. There was a significant age effect for arteriolar (orange) (**b**) and venular (blue) (**c**) amyloid severity score in the cingulate, motor, and somatosensory cortices (p < 0.0001). Alpha = 0.05. The values of the bar graphs were labelled as the mean of five replicates ± sem. (*p ≤ 0.05, **p ≤ 0.01, ***p ≤ 0.001, ****p < 0.0001).

To investigate the relationship between arteriolar and venular amyloid accumulation with disease progression, vascular amyloid load was quantified as percent amyloid coverage of each vessel type (Fig. 3) and amyloid severity score (Fig. 4b, c). Vascular amyloid has been demonstrated to be primarily in the cortex in the TgF344-AD at 9 months of age^1^. As such, average percent amyloid coverage of each vessel was quantified by calculating the area of ThioS immunolabeling over the area of lectin labeling in the cortex, specifically the cingulate, motor, and somatosensory cortices combined (Fig. 3). A significant age effect was observed for both arteriolar (Fig. 3a; p = 0.0001) and venular amyloid (Fig. 3b; p = 0.004); with significantly more arteriolar amyloid compared to venular amyloid at all ages (Fig. 3c; 7 months p = 0.0006, 10 months p = 0.002, 13 months p = 0.0008, 16 months p = 0.002). The progression of amyloid across age varied, where arteriolar amyloid coverage increased from 23.0 ± 3.1% to 31.4 ± 1.3% (mean ± sem, p = 0.02) and venular amyloid coverage increased from 5.8 ± 2.0% to 14.1 ± 1.9% (p = 0.050) between 7 and 10 months of age. There were no significant differences in amyloid coverage between 10 and 16 months of age for both vessel types (Fig. 3a, b). At 7 months, the scatter plot shows high variability in arteriolar and venular amyloid coverage between animals within each cohort, which decreases in arterioles relative to venules as amyloid accumulates (Fig. 3c). These data support previous literature demonstrating increase in CAA with disease progression^23^ as well as it suggests that venular amyloid accumulates in conjunction with severe arteriolar amyloid deposition and that arteriolar and venular amyloid may accumulate at different rates.

The rate of arteriolar and venular amyloid accumulation did not progress linearly, as demonstrated by diminishing differences in arteriolar and venular amyloid coverage across age (Fig. 4a). A one-phase association curve was applied to describe the rate of accumulation and a significant difference was detected in arteriolar and venular amyloid accumulation rates (p < 0.0001). The k-value, a constant represented as inverse time, indicates the rate of arteriolar amyloid (k = 0.439) and venular amyloid (k = 0.386) accumulation (Fig. 4a). These values demonstrate that arteriolar amyloid accumulates faster than venular amyloid (Fig. 4a). The x-intercepts approximate the initiation of amyloid deposition for each vessel type and indicated that arteriolar amyloid initiated at ~ 4.5 months while venular amyloid was closer to 6 months of age (Fig. 4a). Overall, the one-phase association model corroborated the histopathological analyses, demonstrating that venular amyloid accumulates after and to a lesser extent than arteriolar amyloid accumulation. To further explore amyloid accumulation, arteriolar and venular amyloid severity scores were analysed in three distinct areas of the cerebral cortex; the cingulate, motor, and somatosensory cortices (Fig. 4b, c). This semi-quantitative tool allows for an accurate sampling as the analysis can be applied to the entire region of interest rather than an individual vessel-wise analyses. In addition, the scoring system offers the ability to categorize vascular amyloid severity into five defined groups^36,37^. All three brain regions accumulated amyloid with initial accumulation in the somatosensory cortex followed by the motor and finally the cingulate cortices (Fig. 4b, c). Despite a non-significant difference in percent vascular amyloid coverage from 13 to 16-months of age in arteriolar and venular amyloid (Fig. 3a, b), venular amyloid severity score demonstrated a statistically significant difference in the cingulate cortex (p = 0.005), and a positive trend toward significance in the motor (p = 0.05) and somatosensory cortices (p = 0.06). Our data support the role of venular amyloid contributions to the age-dependent vascular amyloidosis.

## Discussion

We observed Aβ deposits first in parenchymal plaques, then as disease progresses, in arteriolar and venular amyloid deposits. The presence of these venular amyloid deposits is not exclusive to the TgF344-AD rats, as venular Aβ also been reported in the APP + PS1 rat^22^ and a modified APP/PS1 mouse^10^, which supports our findings. To determine the interplay between arteriolar and venular amyloid accumulation, we examined histopathological analyses of four age cohorts of TgF344-AD rats, starting early in the disease at 7 months of age up to 16 months of age, with severe CAA deficits and behavioural dysfunction^7,23,38–40^. Histopathological analyses of vascular amyloid showed a significant age effect for both arteriolar and venular accumulation, with more arteriolar amyloid accumulating than venular amyloid within each age group. Moreover, venular amyloid continues to increase significantly later in the disease, when comparing between 13 and 16 months of age. Our data proposes a model that once arterioles become saturated with amyloid, periarterial drainage is significantly impaired leading to a slower rate of amyloid accumulation in arterioles. Perivenular drainage may then compensate, until amyloid deposition within venules results in the dysfunction of perivenular drainage.

ThioS is commonly used to characterize vascular amyloid, yet it does not exclusively target the Aβ peptide, therefore confirmation of vascular Aβ localization was achieved using five Aβ specific antibodies targeting different residues throughout the Aβ peptide sequence. The pattern and localization of arteriolar amyloid reported herein are consistent with previous findings; Aβ aggregates along the outer edge of the vessel, suggesting build-up first along the periarterial spaces^20,21^, and then eventually along the vascular smooth muscle cells to form the ring-like structures^2^. In contrast, venular Aβ deposits are small and globular which increase in size and number with disease severity. We speculate that these deposits could adhere to the mural cells of the venules^41^ or to the aberrant collagen layer^4,14^. In addition, high resolution images demonstrated amyloid deposits that appear cellular in shape, which could represent endocytosed venular amyloid within monocytes, as previously described^10^.

Arteriolar and venular Aβ immunostaining demonstrate more Aβ_42_ staining than Aβ_40_. Previous literature demonstrates higher Aβ_40_: Aβ_42_ ratio in vascular amyloid compared to parenchymal plaques in patients^9^. However, other studies have suggested that Aβ_42_ is the first to be deposited in vascular amyloid and may be important in initiating vascular amyloidosis^27,28^. Since the production of Aβ_42_ increases over Aβ_40_ in the model utilized herein, the difference in the composition of vascular deposits may be a function of model and not disease. Future studies analyzing the Aβ_40_: Aβ_42_ in arterioles and venules would highlight differences in the composition of Aβ peptides present between these vessel types.

Deposition of vascular Aβ along the perivascular spaces of cortical arterioles and venules suggests that impairment of the periarterial and glymphatic drainage is involved in AD and CAA. Venular pathology is important to understanding AD as venular amyloid may contribute to the cerebrovascular dysfunction that occurs in AD. Furthermore, venular Aβ deposition may contribute to venous collagenosis^16,42–44^, increased vascular resistance along the venular walls^14,22,24^, and enlargements of perivenous spaces as amyloid builds-up^8,11,22,46,47^ in AD patients. Overall, these pathologies may produce a positive feedback loop, leading to continued Aβ deposition, impaired perivascular clearance, and further exacerbation of cerebrovascular dysfunction. As these impairments continue to occur in CAA even in the absence of AD^4,9,14^, we speculate that vascular amyloidosis would follow a similar pattern to those presented herein, with severe arteriolar amyloid accumulating and venular amyloid deposits sparingly. With limited research on venular amyloidosis in CAA models^4^, there is a need for future studies examining the interplay between arteriolar and venular Aβ accumulation in pure CAA disease progression. We also highlight that despite limited venular Aβ deposits, it may contribute to the exacerbation of AD and CAA, which we have previously reviewed^4^. An important consideration is that humans have less venules than arterioles^16^, suggesting a greater susceptibility for venular compromise, and contribution to disease progression.

This is the first study to examine arteriolar and venular amyloid throughout disease progression. We demonstrated the progressive accumulation of venular amyloid that continues in the later stage of AD, which proposes a downstream alteration in the perivascular clearance of Aβ shifting from periarteriolar clearance to the glymphatic pathway.

Although these results are novel, some limitations exist with the methodologies utilized. As one sample was provided for each amyloid in the LCM-MS study, it allowed definitive identification of Aβ within amyloid samples, however, comparative analysis between each sample could only be described in terms of relative abundance. Furthermore, with Aβ being highly aggregatory in the amyloid deposits, it posits a challenge in the digestion and extraction of fragments for MS analysis. In addition, due to limitations in breeding colonies, the rats in each age cohort were not sex-balanced and therefore analysis of sex differences were not performed. Moreover, histopathological analysis was a cross-sectional design, only allowing a static description of arteriolar and venular amyloid rather than viewing the relationship over a period of time.

Despite these limitations, this study invites further investigation into the interaction of arteriolar and venular amyloid and how they are distinctly impacted. Moreover, presence of ApoE in both the arteriolar and venular amyloid encourages further investigation into the contributions of ApoE in the clearance of Aβ along both the periarteriolar and venular pathways. With their involvement in the clearance of vascular amyloid^48^, studies examining the mechanisms involved in monocyte/macrophage recruitment and phagocytosis of amyloid would be of great import in finding alternative methods in the treatment of CAA and AD. These studies highlight the importance of understanding the involvement of the cerebral vasculature in AD when studying potential therapeutic strategies in clearing amyloid.

In conclusion, a distinct morphology was observed between arteriolar and venular amyloid, with venular amyloid aggregates being more globular in shape, increasing in size and number with disease progression. The application of the laser capture microdissection with mass spectrometry demonstrated the presence of Aβ in venular amyloid, but to a lesser extent compared to arteriolar amyloid and parenchymal plaques. This in conjunction with the data presented herein suggests significant accumulation of both arteriolar and venular Aβ occurring at different rates. Histopathological analysis further demonstrates presence of significant arteriolar amyloid accumulation relative to venular amyloid deposits withing each age group. Significant vascular amyloid severity in the venules occurs between 13 and 16 months of age, suggesting that venular amyloid accumulation plays a role in vascular amyloidosis at a later stage of disease. Overall, this study demonstrates the need for evaluation of the entire vascular bed’s contribution to disease progression. Recognizing differences between arteriolar and venular pathology may be beneficial in increasing treatment specificity and efficacy, and gaining further understanding of AD progression.

## Methods

### Animals

TgF344-AD rats express two transgenes, APPswe and presenilin 1 with the PS1ΔE9 mutation, driven by the mouse prion promoter on the F344 rat strain^23^. Four cohorts of rats, were utilized; 7 months (3 females, 2 males), 10 months (4 females, 1 male), 13 months (1 female, 4 males), and 16 months (5 females) of age, with a total of 20 rats utilized for this study (n = 5 for each cohort). Non-transgenic rats do not develop vascular amyloid and thus were not analyzed. All rats were bred in-house, and kept under a 12-h light/dark cycle, with an ad libitum access to food and water. All procedures were approved by The Animal Care Committee of the Sunnybrook Research Institute. In addition, experimental procedures adhered to the standards and guidelines of the Canadian Council of Animal Care, and complied with the requirements of the Regulations of the Animals for Research Act. This study is reported in accordance with the Animal Research: Reporting of In Vivo Experiments guidelines.

### Immunofluorescence staining

For brain collection, rats were euthanized by transcardial perfusion with 1xphosphate-buffered-saline containing 0.1% heparin and fixed-perfused using 4% paraformaldehyde. The fixed-perfused TgF344-AD rat brains were coronally sectioned at 40 μm and five sections per rat were selected between 1.28 mm and − 3.24 mm from Bregma. All sections were washed with phosphate-buffered-saline three times for 10 min, submerged in 1% ThioS (Sigma-Aldrich, Canada, 1326-12-1) for 7 min, then washed with 70% ethanol. After three washes with 1 × phosphate-buffered saline, sections were blocked in 5% goat serum and 0.5% triton in 1 × phosphate-buffered saline for one hour. Primary antibody incubation overnight with rabbit anti-alpha smooth muscle actin antibody (1:1000) (Abcam, United Kingdom, ab5694) was conducted, the following day the sections were washed again with 1 × phosphate-buffered saline and incubated with Alexa Fluor 594-conjugated goat-anti-rabbit secondary antibody (1:200) with lycopersicon esculentum lectin (1:200), DyLight 649 (Lectin) (Vector Laboratories, USA, DL-1178). Alternatively, 40 μm sections were washed three times with 1 × tris-buffered saline for 10 min each, treated with 88% formic acid for eight minutes at room temperature (RT), followed by three 10 min washes of 1 × tris-buffered saline with 0.25% triton and a one-hour block incubation containing 2% goat serum and 0.5% bovine serum albumin in 1 × tris-buffered saline with 0.25% triton. Overnight primary antibody incubation was performed with rabbit anti-alpha smooth muscle actin antibody (1:1000) and one of five anti-Aβ antibodies (1:200 each); MOAB-2 (Abcam, U.K, ab126649), 4G8 (Biolegend, USA, 800701), 6E10 (Biolegend, USA, 803015), Aβ_40_ (Biolegend, USA, 805401), and Aβ_42_ (Biolegend, USA, 805501), followed by one of Alexa Fluor 488-conjugated goat anti-rabbit (1:200, Invitrogen, USA, A11008), Lectin 649 (1:200), and either the Alexa Fluor 555-conjugated goat anti-mouse IgG2b antibody (1:200, Invitrogen, USA, A21147) or Alexa Fluor 594-conjugated goat anti-mouse IgG antibody (1:200, Invitrogen, USA, A11032).

### Image analyses

For every transgenic rat, a total of 25 cortical penetrating arterioles and 25 cortical ascending venules (diameter > 10 μm each) within the cingulate, motor, and somatosensory cortices were randomly selected with n = 5 rats per cohort, thus analyzing 125 vessels to obtain percent vascular amyloid of each vessel type. As established by previous reports^1–3,21^, arterioles were defined as containing a structured alpha smooth muscle actin, exhibiting fewer branching and having a uniform diameter along its length while venules were designated to vessels that showed more branching, a larger diameter that increased toward the cortical surface, and lacked the typical smooth structure of the alpha smooth muscle actin. Separating the arterioles and venules, percent vascular amyloid coverage was calculated using ImageJ by measuring area of ThioS+ amyloid staining normalized to the total vessel wall area covered by lectin. The pixel density was established after thresholding background to 7–10 pixels per 150 μm^2^^1,3^. Vascular amyloid was evaluated semi-quantitatively by categorizing penetrating arterioles and ascending venules through five different grades based on severity—no coverage (grade 0), trace/scattered coverage (grade 1), mild (grade 2), moderate (grade 3), and severe coverage (grade 4)—as previously established^36,37^. The distinction in severity scores between arteriolar and venular amyloid across each score have been imaged for reference (Supplementary Fig. S2).

### Laser capture microdissection

For laser capture microdissection two female rats, one at 12 and one at 16 months of age were utilized. Both rats were euthanized using transcardial perfusion with 1xphosphate-buffered saline containing 0.1% heparin. The 12-month-old rat was fixed-perfused with 4% paraformaldehyde while the 16-month-old rat brain was flash frozen in order to maximize venular amyloid acquisition. Rat brains were coronally sectioned at 10 μm (Bregma 1.20 mm through − 6.04 mm), and stained with ThioS and alpha-smooth muscle actin using the same protocol that were applied to the tissues utilized for histopathological analysis. ThioS is an antibody-free dye that stains amyloid aggregates. These sections were then dehydrated using a gradient of ethanol, followed by xylene, airdried, and stored at 4 °C until use. The PALM microbeam microscope, together with the PALM RoboSoftware (Carl Zeiss Microscopy, Germany) was utilized to perform the laser capture microdissection (LCM). Using a freehand selection tool, three distinct amyloid aggregates were cut and isolated; parenchymal plaques (n = 1600 elements), arteriolar amyloid (n = 1900), and venular amyloid (n = 7700). After amyloid acquisition, the tissue was thoroughly inspected along the z-plane to ensure clearance of the ThioS + deposits in both the parenchyma and vasculature. Control samples were collected by isolating the non-plaque associated parenchyma (n = 850), arteriole (n = 1080) and venule (n = 3600) tissue devoid of vascular amyloid. Pooled samples were collected onto AdhesiveCap 500 centrifuge tubes (Carl Zeiss Microscopy, Germany), which were stored at -80 °C prior to mass-spectrometry. LCM vascular amyloid samples were acquired from the cingulate, motor, and somatosensory cortices and the parenchymal plaques were collected in the same regions as well as the entorhinal cortex.

### Mass spectrometry

Proteins were extracted in 8 M urea in 50 mM TRIS (pH = 8.0) for 20 min at RT. Each sample was reduced for 1 h in 10 mM ditiothreitol (DTT) at 60 °C and alkylated 20 mM iodoacetamide for 45 min at RT. To decrease the concentration of urea to < 1 M, 50 mM ammonium bicarbonate was added. The samples were digested overnight using Trypsin (Pierce, Thermo Fisher Scientific, USA) at 37 °C, then lyophilized on a SpeedVac (Thermo Fisher Scientific, USA), desalted using C-18 ziptip (Millipore, Canada), and lyophilized again. The peptides were resuspended in 0.1% formic acid and were separated using liquid chromatography, performed by an Easy-nLC 1200 nano-LC system (Thermo Fisher Scientific, USA) containing one analytical column comprising of the stationary phase, 2 μM C18 beads (Thermo Fisher Scientific, USA). The fragments were loaded onto the analytical column at a maximum pressure of 900 Bar at 60 °C and were fractionated using a linear gradient containing 0.1% formic acid to 80% acetonitrile with 0.1% formic acid (Buffer B) at a flow rate of 250 nL/min. Eluted peptides were ionized with the Nanospray Flex ion source (Thermo Fisher Scientific, USA) and tandem mass spectrometry was completed by the Thermo Scientific Orbitrap Fusion Lumos Tribrid mass spectrometer. A full mass spectra was performed in the Orbitrap mass analyzer at a resolution of 120,000 FWHM and a dynamic exclusion filter was conducted, excluding ions with a mass tolerance window of 10 ppm for 10 s. Ions exceeding the minimum intensity threshold of 10 000 counts were selected for a data-dependent collision-induced ion trap (centroid datatype) fragmentation with a scan range of 200–1400 m/z and a collision energy set at 30%. The tandem mass spectrometry data were analysed against the Uniprot protein sequence FASTA files of the rat proteome with the addition of the human APP sequence. The MS-Amanda Proteome Discoverer (Research Institute of Molecular Pathology, Austria), Sequest (XCorr Only) (Thermo Fisher Scientific, USA), and X! Tandem (The GPM, thegpm.org) are the three databases used for the analysis and were searched with a fragment ion mass tolerance of 0.60 Da. Validation was completed by the Scaffold Software (version 4.10.0, Proteome Software Inc., USA). Any peptides exceeding a 95.0% probability threshold, established by the Peptide Prophet algorithm^50^, and had at least two fragments identified were reported.

### Statistical analysis

Statistical analysis was performed using GraphPad Prism (Version 6.01, Graphpad Prism, USA) and data was described as the mean ± standard error of the mean (SEM). A one-way analysis of variance with a Holm-Sidak post hoc test was performed to analyze percent vascular amyloid coverage of arterioles and venules across all four age groups independently to allow for multiple comparisons (n = 5 per age cohort). Paired two-tailed t-tests were performed to compare percent arteriolar and venular amyloid within an age cohort (n = 5 per age cohort). The rate of arteriolar and venular amyloid accumulation was assessed using a nonlinear regression analysis. Separate one-way ANOVAs were used to analyze the proportion of amyloid-positive arterioles and venules in three regions of the cortex with disease progression; somatosensory, motor, and the cingulate cortices (n = 5 per age cohort). No data were excluded in our results.

### Ethics declaration

All experimental procedures were dually approved and adhered to the guidelines established by The Animal Care Committee of The Sunnybrook Research Institute and the Canadian Council of Animal Care. In addition, animal experiments complied with the standards of the Regulations of the Animals Research Act. No human participants were used for this study. This study is reported in accordance with the Animal Research: Reporting of In Vivo Experiments guidelines.

## Supplementary Information

Supplementary Information 1.

Supplementary Information 2.

Supplementary Information 3.

Supplementary Information 4.

Supplementary Information 5.

## Data Availability

All data analysed during this study are included in the published article [and its supplementary information files].

## Abbreviations

Aβ: Amyloid-beta peptide
AD: Alzheimer’s disease
ApoE: Apolipoprotein E
APP_swe_: Swedish amyloid precursor protein mutation
CAA: Cerebral amyloid angiopathy
PS1ΔE9: Presenilin 1 with exon 9 excision
ThioS: Thioflavine S
