# Supplementary material for: Venular amyloid accumulation in transgenic Fischer 344 Alzheimer’s disease rats
Source: Sci Rep. 2022 Sep 10;12:15287. doi: 10.1038/s41598-022-19549-y (PMC9464208; doi:10.1038/s41598-022-19549-y)
Supplement: Supplementary file 1 — Supplementary Information 1. [file 41598_2022_19549_MOESM1_ESM.pdf]

Venular amyloid accumulation subsequent to cerebral amyloid angiopathy in transgenic Fischer 344 Alzheimer's disease rats

J. Bishay[1], T.L. Beckett[1], A.Y. Lai[1], M.E. Hill[1], D. McMahon[2], J. McLaurin*[1,3].

[1]Biological Sciences; Sunnybrook Research Institute; Toronto, ON, M4N 3M5; Canada. [2]Physical Sciences; Sunnybrook Research Institute; Toronto, ON, M4N 3M5; Canada. [3]Laboratory Medicine and Pathobiology, Temerty Faculty of Medicine; University of Toronto; Toronto, ON, M5S 1A8; Canada.

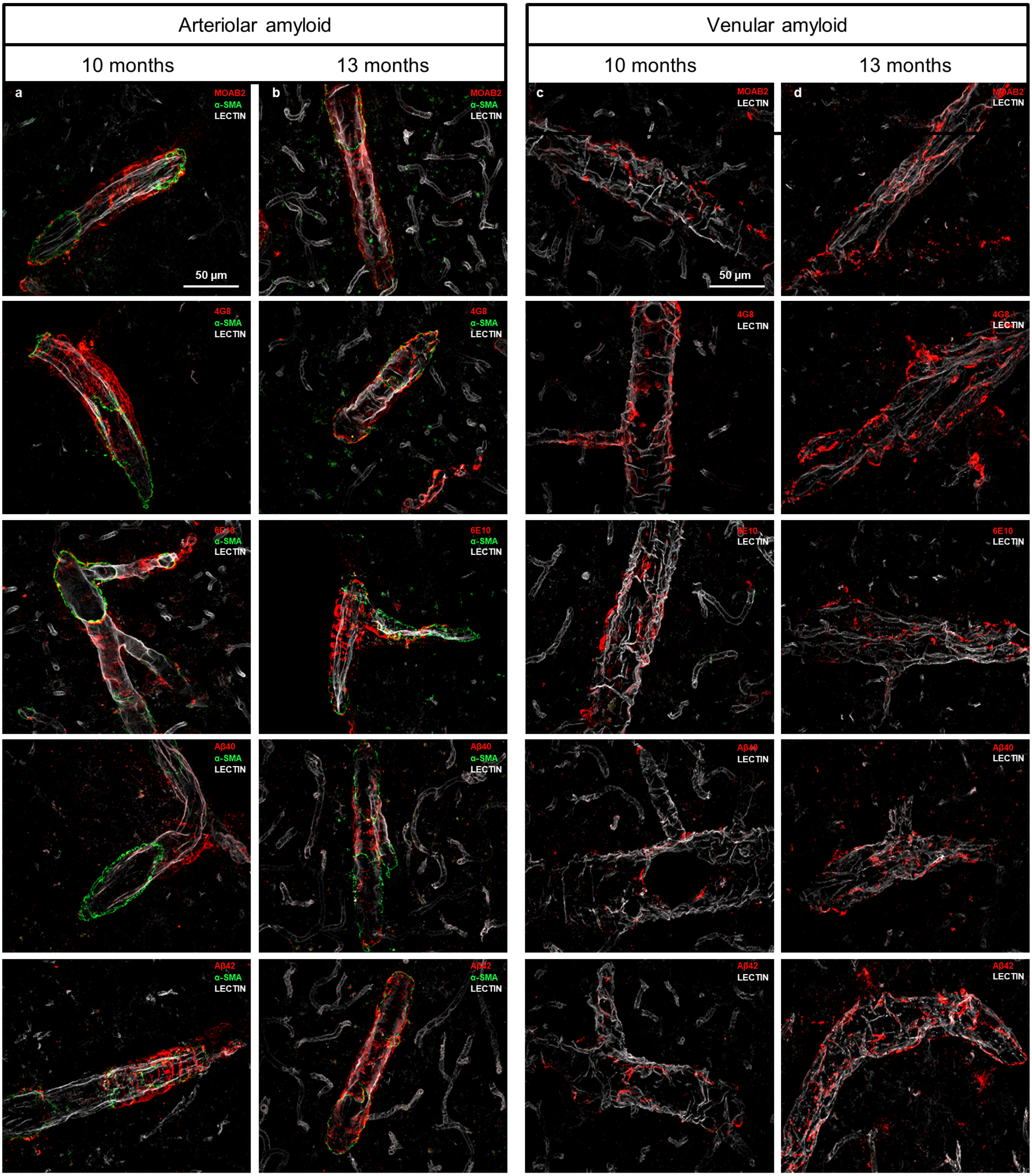

Supplementary Figure S1. Arteriolar and venular amyloid deposition at 10 and 13 months of age. An array of Aβ antibodies, including MOAB2, 4G8, 6E10, $Aβ_{40}$, and $Aβ_{42}$ (red) were used to visualize arteriolar **(a, b)** and venular **(c, d)** Aβ. Vascular Aβ at 10 months **(a, c)** and 13 months **(b, d)** demonstrated progression of arteriolar and venular Aβ. High resolution images were obtained at a 40x objective.

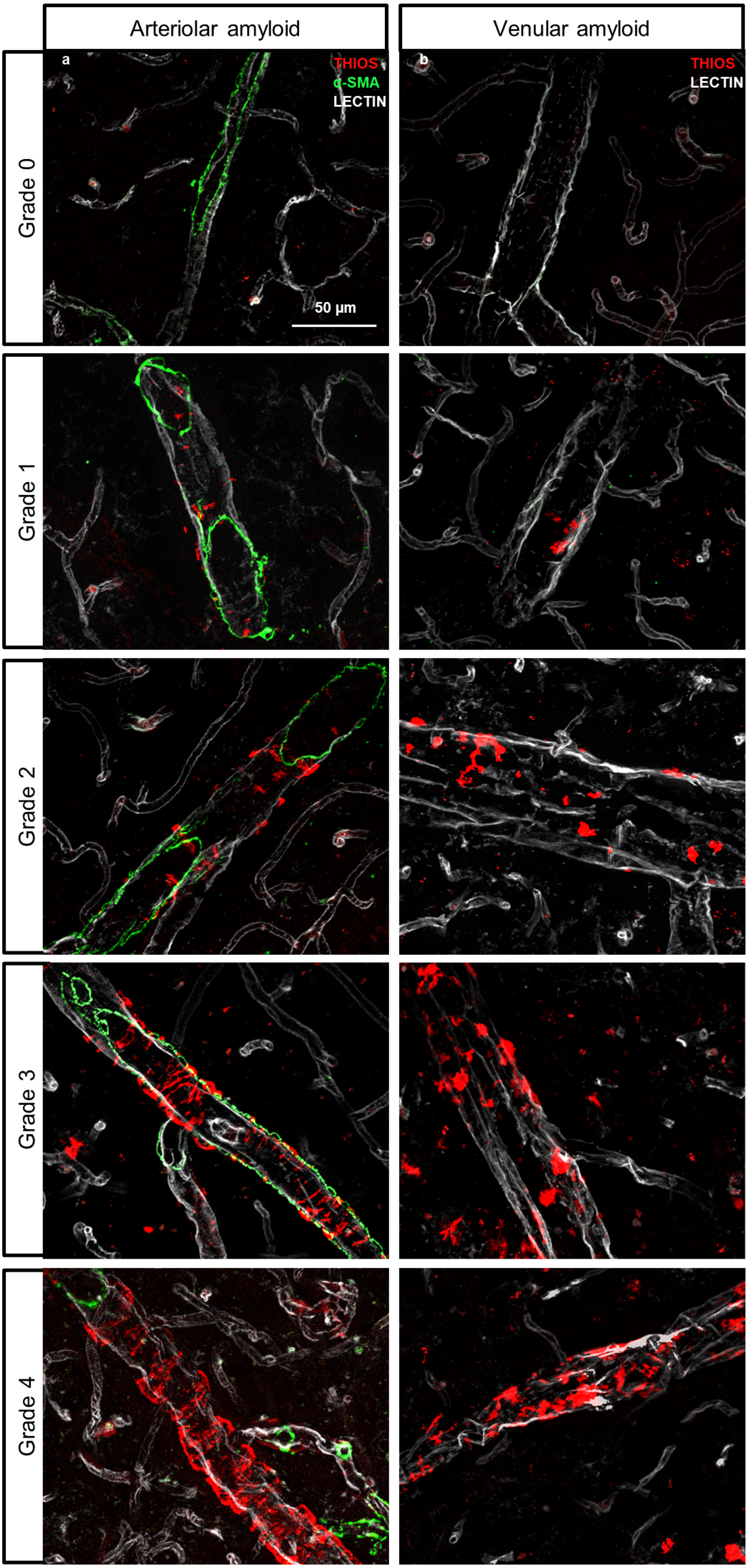

Supplementary Figure S2. Vascular Amyloid Severity Score Classification. Vascular amyloid was evaluated semi-quantitatively by categorizing penetrating arterioles and ascending venules through five different grades – no coverage (grade 0), trace/scattered coverage (grade 1), mild (grade 2), moderate (grade 3), and severe coverage (grade 4) – as established by previous literature [36, 37]. For arteriolar amyloid **(a)**, a score of 2 represented partial circumferential staining, a score of 3 indicated an almost complete circumferential staining, and a score of 4 displayed complete circumferential staining with amyloid spreading into the surrounding neuropil. A score of 2 for venular amyloid **(b)** indicated by an increase in the number of small globular deposits compared to a score of 1. A score of 3 indicated further accumulation of the globular deposits which are less scattered, with a presence of slightly larger deposits. Severe coverage (score = 4) is represented by substantial aggregation of larger sized globular deposits around the venule. Images were acquired with a 20x objective.
